# McGill University, Comparative Psychology Society

**Published:** 1896-05

**Authors:** 


### SOCIETY PROCEEDINGS.

### PROCEEDINGS FOR THE YEAR 1894–95 OF THE SOCIETY FOR THE STUDY OF COMPARATIVE PSYCHOLOGY IN CONNECTION WITH THE FACULTY OF COMPARATIVE MEDICINE AND VETERINARY SCIENCE OF McGILL UNIVERSITY, MONTREAL.

(Continued from page 347.)

Mr. J. C. Hargrave followed with a paper on "Feigning Death," which habit is resorted to for various reasons by many animals, even as low in the scale as the insecta, and the simulation of death cannot always be considered a contrivance of cunning, being frequently the consequence of terror. Violent stimuli will produce a disorganization of nerve centres, so that nervous control is almost entirely in abeyance, and there may even be a complete cessation of vital activities and consequent death. Preyer ascribes the shamming of death as seen in insects to the exclusive influence of cataplexy, having observed even in crayfish the potentiality of this influence on the neuro-muscular system; but the fact should be borne in mind that no species of the insecta when shamming really assumes the attitude of death, so that shamming in these cases may be considered to be "an instinctive action to remain as motionless and inconspicuous as possible, and which has been developed in conformity with the law of natural selection," and in this opinion so great a student as Romanes concurs. This instinct we find well developed, along with protective resemblance in those animals which are not adapted for rapid flight from enemies. But this explanation will not hold good in the case of some of the higher animals, as for instance, the fox, and we have evidence that some animals feign death for the purpose not of escaping, but to deceive other animals. An instance was related of a pet bear, owned in the writer's family, which was in the habit of shamming to enable him the more easily to catch unwary fowls, and the same habit has been frequently reported as obtaining among monkeys. Romanes mentions the case of a captive monkey who, having been annoyed by crows stealing his rations, appeared one morning as if seriously indisposed, closing his eyes, drooping his head, and exhibiting other symptoms of illness. The crows taking advantage of his seemingly indifference approached with impunity, but no sooner had they come within reach than all signs of illness vanished and the cunning animal seized one unlucky bird and proceeded to pluck it with the most humorous gravity, and having completed the task threw the bird away. From this it would seem that the animal had a knowledge of what sickness meant, having probably observed other animals, and reasoned that simulation would be of service to him, and this theory of utility would apply to other vertebrate animals. As already shown, feigning may be caused by fright, the stimuli producing a loss of the power of coordination, and the animal remains motionless, so that the habit in general cannot be said to be due to any one cause in particular.

In the discussion which followed the reading of these papers, Mr. Zink asked what relation consciousness bore to the dominant and constituent parts. The President replied that consciousness was the direct outcome of the molecular constitution of an organism. It is special arrangement that gives rise to consciousness; that is, there may be simpler forms than give rise to consciousness, but which develop into it.

An article on the "Psychology of Hypnotism," by Dr. Weir, and which recently appeared in the *American Naturalist*, was read by one of the members. The writer describes three stages of consciousness, viz., subconsciousness, inco-ordinate, and co-ordinate consciousness, and defined hypnosis as "a brown study accomplished by neurotic degenerations," but owing to the lateness of the hour there was no discussion on the subject.

The minutes of the previous meeting were read and adopted, and the addition of several new volumes to the Library was announced.

Mr. Alex. Cowan read a paper on "Æstheticism and Religion in Animals," first comparing the views of Huxley, Wilkes, and Herbert on the differences between man and the lower animals, and showing that some of the physical attributes of man are inferior to corresponding ones in animals. "His teeth," says Prof. Cope, "are thoroughly primitive." Compare his power of sight with that of the eagle or his olfactory sense with that of the dog, and one is forced to admit that in some respects, at least, the lower animals are superior to man. The progressive period of mental development of the monkey is short, and at an early age becomes stationary, but the same condition is seen, for example, in the negro, whose mental faculties reach the maximum point of development sooner than in the Caucasian. In ratio to the development of the hand we find appreciation of the beautiful, but, as shown by Darwin, there are animals without hands who also share in the æsthetic sense. Birds take pleasure in brilliant plumage and sweet sounds, and the feeling thus awakened plays an important part in the preservation and perfection of species through natural selection. "The decoration of its boudoir" by the bower bird would indicate the possession of this sense in a marked degree. The so-called bower is built of sticks, and adorned with feathers, shells, colored rags, and anything of bright color which may please the bird's fancy. Some of these objects are brought a considerable distance, and are of no use but to gratify the æsthetic tastes of the occupant; and this bower is not a nest in which eggs are laid or young ones reared, but serves as a salon for entertainment. The singing of birds implies a certain appreciation of melody, and dogs are by no means indifferent to the effect of music. The fertilization of many plants depends on the existence of color-sense in insects. Even the religious sentiment is faintly shadowed in the lower animals, who seek to win favor of those whom they regard as superiors, fawning and grovelling in adoration, or to crave pardon for some offence. One writer has said there is no difference between the negro who worships an animal and the dog that crouches at his master's feet. Animals fly to man for protection, as a believer does to his God. "This feeling of religious devotion," says Darwin, "is a highly complex one, consisting of love, complete submission to an exalted and mysterious superior, a strong sense of reverence, dependence, fear, hope, gratitude, and perhaps other elements. No being could experience so complex an emotion until advanced in intellectual and moral faculties to, at least, a moderately high level. Nevertheless we see some distinct approach to this state of mind in the deep love of a dog for his master, associated with complete submission, fear, and perhaps other feelings." Romanes one day tied a long thread to a bone, which he gave to a Skye terrier to play with. He says: "After he had tossed it about for a short time I took the opportunity, when it had fallen a short distance from him, of gently drawing it away by means of the thread. Instantly his whole demeanor changed. His astonishment knew no bounds. He first approached it with nervous caution, but, as the slow receding motion continued and he became certain that the movement could not be produced by any force which he himself had exerted, his astonishment developed into fear, and he ran to conceal himself and watch at a distance the 'uncanny' spectacle." In this instance we have close observation, judgment, reason, and imagination, ending in the exhibition of superstitious fear—all the elements, in short, which constitute religious sentiment in its crudest form. Animals are afraid of darkness, also thunder, lightning, and other meteorological phenomena which inspire primitive man with awe, simply because they are of mysterious origin.

(To be continued.)

